# Posterior Interosseous Nerve Syndrome due to Schwannoma – A Case Report

**DOI:** 10.1055/s-0042-1749614

**Published:** 2022-10-14

**Authors:** Antônio Lourenço Severo, Deodoro Máximo de Alencar Neto, Marcelo Barreto Lemos, Matheus Predebon Duarte, Ivânio Tagliari

**Affiliations:** 1 Serviço de Cirurgia e Microcirurgia da Mão no Instituto de Ortopedia e Traumatologia da Universidade Federal da Fronteira Sul, HSVP - Hospital São Vicente de Paulo, Passo Fundo, RS, Brasil; 2 Projetos e Processos de Fabricação, Universidade de Passo Fundo, Passo Fundo, RS, Brasil

**Keywords:** nerve compression syndromes, neurilemmoma, peripheral nervous system neoplasms, radial nerve, radial neuropathy

## Abstract

Posterior interosseous nerve syndrome is the most frequent syndrome of radial nerve compression, with the arcade of Frohse being the main site of compression. Its symptoms include difficulties in finger and wrist extension with possible radial deviation. Herein, we present a case of posterior interosseous syndrome caused by a schwannoma, a type of neurological tumor.

## Introduction

The radial nerve originates from the posterior fascicle of the brachial plexus, innervating all the posterior compartment muscles of the arm and forearm. Particularly, radial nerve compression in the proximal region of the forearm can result in posterior interosseous nerve syndrome, which specifically affects the extensor compartment muscles of the forearm. 1

However, posterior interosseous syndrome may present different signs and symptoms with varying degrees of intensity. Therefore, history taking and physical examination are crucial for its diagnosis, and electroneuromyography is an important complementary test to confirm diagnosis.

The objective of this report was to describe the symptoms and surgical procedure for posterior interosseous nerve syndrome caused by a schwannoma (also called neurilemmoma), which is a type of peripheral nervous system tumor.

## Case Report

A 65-year-old female patient, who was a professor of exact sciences, presented with a chief complaint of pain at the end of the day, which worsened with the loss of extension movement in the third, fourth, and fifth fingers of her left hand. This condition progressed for 2 months and was associated with sporadic paresthesia in the dorsum of the left hand and forearm. On physical examination, she reported pain on palpation of the proximal and radial third of the left forearm adjacent to the radial head; however, no tumor mass was palpated or observed. A radiography was requested for differential diagnosis without showing abnormalities.

Posterior interosseous nerve syndrome was then diagnosed on electroneuromyography, and a surgical procedure was performed. The patient was asked if data concerning the case could be submitted for publication, and she consented.

**Fig. 1:**
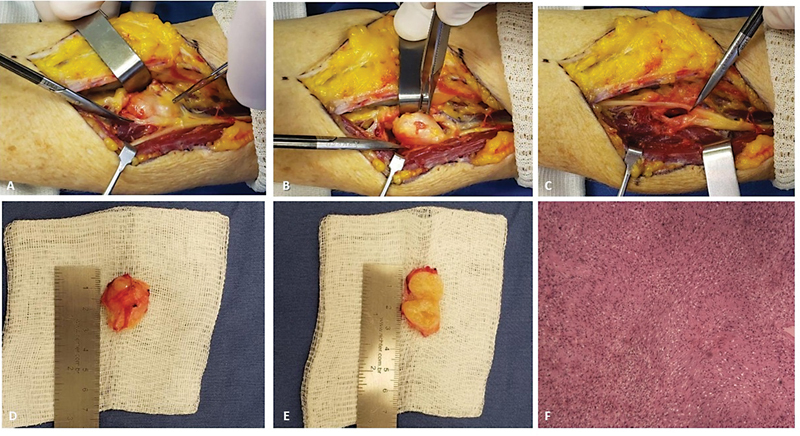
( **A** ) Intraoperative photographs of the anterior forearm approach. ( **B** ) The tumor involving the posterior interosseous nerve. ( **C** ) Tumour resection. ( **D and E** ) The tumor and its cross-section demonstrating fibroelastic and yellowish characteristics. ( **F** ) An anatomopathological image showing spindle-shaped cells, with a concentration of deformed and undulated nuclei as sparse mitoses, which point to a diagnosis of schwannoma.

During the surgical procedure, an incision of ∼ 10 cm was made in the proximal radial third of the left forearm, wherein strangulation of the posterior interosseous nerve at the arcade of Frohse was visualized due to the presence of a 3-cm ^3^ fibroelastic tumor. The mass was then removed, subsequently decompressing the posterior interosseous nerve ( Fig. 1 - A, B, C, D, E, and F). After 6 months and with the help of physical therapy, the patient presented with full motor recovery of her left-hand fingers.

## Discussion

The radial nerve exits the posterior trunk of the brachial plexus, dividing itself into the superficial (sensory) and deep (motor) branches in the proximal forearm, which then innervate all the muscles of the posterior compartment of the forearm. 2

In particular, posterior interosseous nerve syndrome can result from trauma; **expansive lesions, such as tumors** ; local nerve inflammation, such as rheumatoid arthritis; and nonspecific brachial neuritis. Notably, cases of posterior interosseous nerve compression secondary to schwannoma or neurilemmoma are rare, with only a few cases being reported in the literature. Despite being the most common benign tumor of the peripheral nerve sheath, it accounts for only 5% of soft-tissue tumors, with a high incidence in the head and neck. Furthermore, in cases when a schwannoma affects the upper limbs, it has been reported to prefer the anterior surface of the forearm due to the high concentration of nerve fibers, of which the ulnar and median nerves are the most affected. 3

In the case of this patient, radiography was requested for differential diagnosis, and electroneuromyography for confirmation. As there was no palpable mass, imaging tests such as ultrasound (US) or magnetic resonance imaging (MRI) were not requested, since the motor alteration was already triggered. However, some authors, such as Galbiatti et al. 3 and Wheeler and DeCastro 4 believe that, although the clinical examination is paramount, an image exam such as US or MRI should be requested for differential diagnosis of soft tissues.

Such patients may present with weakness in finger extension, and the wrist may deviate radially due to the weakness of the extensor carpi ulnaris muscle. Depending on its severity, a positive digit-digital percussion sign at the site of the lesion can also be elicited. 5

The initial treatment for posterior interosseous nerve syndrome is conservative, including wrist splinting, anti-inflammatory and analgesic therapy, physical therapy, and daily activity changes. 4

**Fig. 2:**
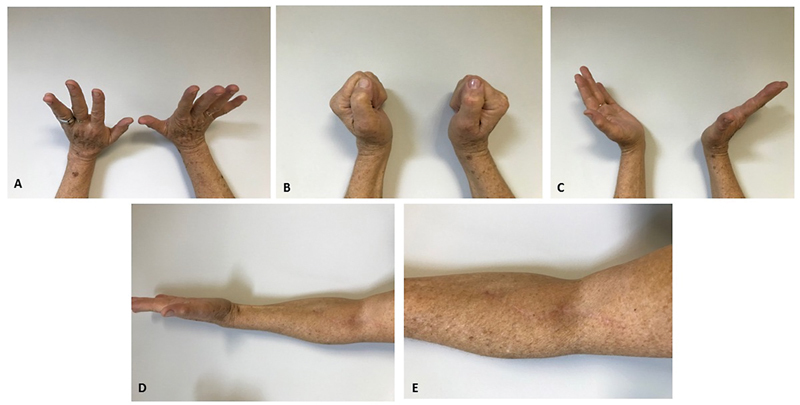
The late postoperative period, after 6 months of physical therapy. ( **A, B** , and **C** ) Images showing wrist and finger extension recovery. ( **D** and **E** ) Photograph of the surgical incision site and its healing.

Surgical treatment is only reserved for cases refractory to conservative management for at least 3 months. Surgical decompression for posterior interosseous nerve syndrome focuses on releasing the areas of compression, including the fibrous bands superficial to the radiocapitelar joint, fibrous border of the extensor carpi radialis brevis, the arcade of Frohse, and the distal border of the supinator. 4 In this case, as there were already motor signs and symptoms, a surgical treatment was immediately chosen. Following surgery, the patient underwent physical therapy, recovering left-hand motor patterns in 6 months ( Fig. 2 ).
