# Supplementary material for: Posterior Interosseous Nerve Syndrome due to Schwannoma – A Case Report
Source: Rev Bras Ortop (Sao Paulo). 2022 Oct 14;59(Suppl 1):e91–3. doi: 10.1055/s-0042-1749614 (PMC11254416; doi:10.1055/s-0042-1749614)
Supplement: Related texts

# Research article: Síndrome interóssea posterior devido a schwannoma – Um relato de caso

**Keywords:** neoplasias do sistema nervoso periférico, nervo radial, neurilemoma, neuropatia radial, síndromes de compressão nervosa

## Abstract

A síndrome interóssea posterior é a síndrome mais frequente de compressão do nervo radial, com a arcada de Frohse sendo o principal local de compressão. Seus sintomas incluem dificuldades na extensão do dedo e punho, com possível desvio radial. Aqui, apresentamos um caso de síndrome interóssea posterior causada por um schwannoma, um tipo de tumor neurológico.

## Introdução

O nervo radial se origina do fascículo posterior do plexo braquial, inervando todos os músculos compartimentares posteriores do braço e do antebraço. Particularmente, a compressão do nervo radial na região proximal do antebraço pode resultar em síndrome nervosa interóssea posterior, que afeta especificamente os músculos extensores do antebraço. 1

No entanto, a síndrome interóssea posterior pode apresentar diferentes sinais e sintomas com diferentes graus de intensidade. Portanto, a realização de anamnese e exame físico são cruciais para seu diagnóstico, e a eletroneuromiografia é um importante teste complementar para confirmá-lo.

O objetivo deste relatório foi descrever os sintomas e procedimento cirúrgico para síndrome nervosa interóssea posterior causada por um schwannoma (também chamado de neurilemoma), que é um tipo de tumor do sistema nervoso periférico.

## Relato de caso

Uma paciente do sexo feminino de 65 anos, professora de ciências exatas, apresentou uma queixa principal de dor ao final do dia, que piorou com a perda do movimento de extensão no terceiro, quarto e quinto dedos de sua mão esquerda. Essa condição progrediu por 2 meses e foi associada à parestesia esporádica no dorso da mão esquerda e no antebraço. No exame físico, ela relatou dor na palpação do terço proximal e radial do antebraço esquerdo adjacente à cabeça radial; no entanto, nenhuma massa tumoral foi palpada ou observada. A radiografia foi solicitada para diagnóstico diferencial sem apresentar anormalidades.

A síndrome interóssea posterior foi então diagnosticada em eletroneurografia, e foi realizado um procedimento cirúrgico. A paciente foi questionada se os dados relativos ao caso poderiam ser submetidos à publicação e ela consentiu.

Durante o procedimento cirúrgico, foi realizada uma incisão de ∼ 10 cm no terço radial proximal do antebraço esquerdo, onde o estrangulamento do nervo interósseo posterior na arcada de Frohse foi visualizado devido à presença de um tumor fibroelástico de 3 cm ^3^ . A massa foi então removida, posteriormente descomprimindo o nervo interósseo posterior ( Fig. 1 - A, B, C, D, E e F). Após 6 meses e com a ajuda da fisioterapia, a paciente apresentou recuperação motora completa dos dedos da mão esquerda.

**Fig. 1:** ( **A** ) Fotografias intraoperatórias da abordagem anterior do antebraço. ( **B** ) O tumor envolvendo o nervo interósseo posterior. ( **C** ) Ressecção do tumor. ( **D e E** ) O tumor e sua seção transversal demonstrando características fibroelásticas e amareladas. ( **F** ) Uma imagem anatomopatológica mostrando células em forma de fuso, com concentração de núcleos deformados e ondulados com mitoses esparsas, que apontam para um diagnóstico de schwannoma.

## Discussão

O nervo radial sai do tronco posterior do plexo braquial, dividindo-se nos ramos superficial (sensorial) e profundo (motor) no antebraço proximal, que então inerva todos os músculos do compartimento posterior do antebraço. 2

Em particular, a síndrome nervosa interóssea posterior pode resultar de traumas; **lesões expansivas, como tumores** ; inflamação nervosa local, como artrite reumatoide; e neurite braquial não específica. Notavelmente, casos de compressão nervosa interóssea posterior secundária ao schwannoma ou neurilemoma são raros, com apenas alguns casos relatados na literatura. Apesar de ser o tumor benigno mais comum da bainha nervosa periférica, ele representa apenas 5% dos tumores de tecido mole, com alta incidência na cabeça e pescoço. Além disso, nos casos em que um schwannoma afeta os membros superiores, tem sido relatado que prefere a superfície anterior do antebraço devido à alta concentração de fibras nervosas, das quais os nervos ulnar e mediano são os mais afetados. 3

No caso desta paciente, foi solicitada uma radiografia para diagnóstico diferencial e eletroneurografia para confirmação. Como não havia massa palpável, não foram solicitados exames de imagem como ultrassom (US) ou ressonância magnética (RM), uma vez que a alteração motora já foi acionada. No entanto, alguns autores, tais como Galbiatti et al. 3 e Wheeler e De Castro, 4 acreditam que, embora o exame clínico seja primordial, um exame de imagem como o US ou a RM deve ser solicitado para diagnóstico diferencial de tecidos moles.

Esses pacientes podem apresentar fraqueza na extensão do dedo e o punho pode desviar-se radialmente devido à fraqueza do músculo extensor carpi ulnaris. Dependendo de sua gravidade, um sinal de percussão digital de dígito positivo no local da lesão também pode ser provocado. 5

O tratamento inicial para síndrome nervosa interóssea posterior é conservador, incluindo imobilização de punho, anti-inflamatório e analgésico, fisioterapia e alterações diárias de atividade. 4

O tratamento cirúrgico só é reservado para casos refratários à gestão conservadora por pelo menos 3 meses. A descompressão cirúrgica para síndrome interóssea posterior concentra-se na liberação das áreas de compressão, incluindo as bandas fibrosas superficiais à articulação radiocapitelar borda fibrosa do extensor carpi radialis brevis, a arcada de Frohse, e a borda distal do supinador. 4 No caso aqui descrito, como já havia sinais e sintomas motores, um tratamento cirúrgico foi imediatamente escolhido. Após a cirurgia, a paciente foi submetida a fisioterapia, recuperando os padrões motores da mão esquerda em 6 meses ( Fig. 2 ).

**Fig. 2:** O período pós-operatório tardio, após 6 meses de fisioterapia. ( **A, B e C** ) Imagens mostrando a recuperação da extensão do pulso e do dedo. ( **D e F** ) Fotografia do local de incisão cirúrgica e sua cura.
